# Interleukin-17 directly stimulates tumor infiltrating Tregs to prevent cancer development

**DOI:** 10.3389/fimmu.2024.1408710

**Published:** 2024-06-14

**Authors:** William C. Theune, Ju Chen, Eileen Victoria Theune, Xiaoyang Ye, Antoine Ménoret, Anthony T. Vella, Kepeng Wang

**Affiliations:** ^1^ Department of Immunology, School of Medicine, University of Connecticut Health Center, Farmington, CT, United States; ^2^ The Eighth Clinical Medical College of Guangzhou University of Chinese Medicine, Foshan Hospital of Traditional Chinese Medicine, Foshan, Guangdong, China

**Keywords:** interleukin-17, regulatory T cells, colorectal cancer, RNA splicing, inflammation

## Abstract

**Background:**

Interleukin-17 (IL-17) family cytokines promote protective inflammation for pathogen resistance, but also facilitate autoimmunity and tumor development. A direct signal of IL-17 to regulatory T cells (Tregs) has not been reported and may help explain these dichotomous responses.

**Methods:**

We generated a conditional knockout of *Il17ra* in Tregs by crossing *Foxp3-YFP-Cre* mice to *Il17ra-flox* mice (*Il17ra* ^ΔTreg^ mice). Subsequently, we adoptively transferred bone marrow cells from *Il17ra* ^ΔTreg^ mice to a mouse model of sporadic colorectal cancer (*Cdx2-Cre* ^+^/*Apc* ^F/+^), to selectively ablate IL-17 direct signaling on Tregs in colorectal cancer. Single cell RNA sequencing and bulk RNA sequencing were performed on purified Tregs from mouse colorectal tumors, and compared to those of human tumor infiltrating Treg cells.

**Results:**

IL-17 Receptor A (IL-17RA) is expressed in Tregs that reside in mouse mesenteric lymph nodes and colon tumors. Ablation of IL-17RA, specifically in Tregs, resulted in increased Th17 cells, and exacerbated tumor development. Mechanistically, tumor-infiltrating Tregs exhibit a unique gene signature that is linked to their activation, maturation, and suppression function, and this signature is in part supported by the direct signaling of IL-17 to Tregs. To study pathways of Treg programming, we found that loss of IL-17RA in tumor Tregs resulted in reduced RNA splicing, and downregulation of several RNA binding proteins that are known to regulate alternative splicing and promote Treg function.

**Conclusion:**

IL-17 directly signals to Tregs and promotes their maturation and function. This signaling pathway constitutes a negative feedback loop that controls cancer-promoting inflammation in CRC.

## 1 Introduction

Chronic inflammation is a key driver of tumor development and contributes to approximately one-fifth of all human cancers (1). Patients with inflammatory bowel diseases (IBDs), such as ulcerative colitis (2) and Crohn’s disease (3) have an increased risk of colorectal cancer (CRC). Considerable evidence has demonstrated that the balance between regulatory T cell (Treg) and effector T cell populations are key to the pathogenesis of IBDs and other autoimmune disorders (4). In particular, the presence of Tregs is important for the maintenance of gut homeostasis and resistance to induced colitis in mice (5). Antibody depletion or genetic ablation of IL-2RA (CD25), a marker and functional moiety of Tregs, leads to reduced Treg number and suppressive function, and the development of lethal autoimmunity in these mice (6, 7). Ablation of IL-10, either globally or specifically in T cells, results in severe colitis and spontaneous development of colonic tumors (8–10). Similarly, loss of *Ebi3*, one of the two chains of dimeric cytokine IL-35, on T cells, induces colitis in mice (11). Treg-specific ablation of IL-35 reduced their suppressive function and rendered them ineffective in ameliorating IBD (12). Tregs are known to suppress Th17-mediated inflammation, as *Ebi3* knockout mice exhibit a significant increase in Th17 cell populations, and recombinant IL-35 treatment reduced Th17 differentiation and function (11). Patients with IBDs confirm that an increase in Th17 cell number occurs commensurate with a reduction in Tregs (13).

Interleukin-17 (IL-17) family cytokines are proinflammatory cytokines that promote tumor development in multiple organs (14). IL-17 members include IL-17A- F and bind to both the canonical IL-17 receptors IL-17RA, B, C, D, and E, as well as the noncanonical receptor CD93 (15, 16). Engagement of IL-17 activates mitogen-activated protein kinases (MAPK), nuclear factor-kappa B (NF-κB) and CCAAT-enhancer binding protein (C/EBP) through adaptor proteins ACT1 and TRAF6 (17, 18). IL-17 family cytokines signal to diverse cellular targets, including epithelial cells, fibroblasts, and myeloid cells (15, 18). In CRC, IL-17 promotes cancer progression in transformed epithelial cells in sporadic and induced colorectal cancer models (19, 20). Ablation of IL-17RA, the common receptor of IL-17 family cytokines, in tumor cells, leads to a significant reduction in the number of tumors within the mouse colon (19, 21). This pro-tumor role of IL-17RA is activated mainly by IL-17A, C, and F, whose ablation reduced tumor development in the gut (20, 22, 23). The direct impact of IL-17 on T cells is less known, except for the signaling of IL-17C through IL-17RE on Th17 cells that potentiates a Th17 response and exacerbates autoimmunity (24). To our knowledge, there is no report on direct signaling of IL-17 on other T cells, such as Tregs. Therefore, the understanding of an IL-17-Treg interaction is limited to the role of Tregs in suppressing inflammation, and the indirect role of IL-17 in regulating Treg function by impacting other cell lineages.

To test if IL-17 directly impacts Treg function, we employed a Treg-specific Cre line *Foxp3-YFP-Cre*, and crossed it to *Il17ra* ^F/F^ mice, to selectively ablate IL-17 signaling in Tregs. Further, we grafted bone marrow cells from these mice to a mouse model of sporadic CRC. Under this scheme and using scRNAseq, we interrogated the distribution of IL-17RA and its co-receptors in Tregs from colorectal tumors and draining lymph nodes, and demonstrated that IL-17 directly signals to tumor-infiltrating Tregs to support their immune suppressive function. This result highlights the potential utility of this axis in the treatment of CRC.

## 2 Materials and methods

### 2.1 Animal models

C57BL/6, *Apc^F/F^* (25), *Cdx2-Cre* (26) and *Foxp3-YFP-Cre* mice were obtained from the Jackson Laboratory. *Il17ra* ^F/F^ mice (19) were obtained from Dr. Michael Karin’s laboratory at the University of California, San Diego.

To generate the mouse model of sporadic CRC, *Cdx2-Cre* and *Apc^F/F^* mice were crossed to generate *Cdx2-Cre^+^*/*Apc* ^F/+^ mice. These mice were sacrificed at around 5 months of age for tumor analyses. The mouse colon was dissected, and colorectal tumors were measured with a caliper and excised with scissors. Tumor load was represented as the sum of the diameters of all tumors from each animal. Statistical significance of tumor number, size, and load was determined by Student’s t test for pairwise comparison, with *p* value < 0.05 considered significant.

All mice were maintained in specific-pathogen-free conditions in filter-topped cages on autoclaved food and water at UConn Health. All experiments used co-housed, gender-matched littermates to ensure the consistency of common microflora. Both male and female mice were used for all experiments. All animal experiments were approved by the IACUC of UConn Health.

### 2.2 Bone marrow transplantation

Six- to eight-week-old recipient mice were irradiated twice in one day to achieve a lethal dose (2 x 600 rad) and intravenously injected with a single-cell suspension of 10^7^ donor bone marrow cells. Recipients were co-housed littermates, which were transplanted with both gene-deficient (*Foxp3-YFP-Cre* ^+^ */Il17ra^F/F^*) and control (*Foxp3-YFP-Cre* ^+^ */Il17ra^F/WT^*) bone marrow for comparison. After transplantation the recipients were placed on sulfamethoxazole and trimethoprim in drinking water for two weeks, followed by regular water. Mice were sacrificed and analyzed for tumor development 3-4 months after transplantation.

### 2.3 Flow cytometry and cell sorting

Mouse colorectal tumors were minced with scissors and digested with 1mg/kg collagenase IV (Sigma Aldrich, Cat # C5138) for 20 minutes. Cells were filtered with a 70-μm cell sieve, and stained with Live/Dead fixable exclusion dye (Tonbo Bioscience, Cat # 13-0868), followed by fluorochrome-conjugated antibodies in PBS with 2% fetal bovine serum (FBS) and 1mM EDTA. Anti-CD3 (Cat # 100206), anti-CD4 (Cat # 100536), anti-CD45 (Cat # 103138), anti-IFN-γ (Cat # 505808) and anti-IL-17A (Cat # 506904) antibodies were from Biolegend. Anti-Foxp3 (Cat # 11-5773-82) antibody was from eBioscience. Anti-CD8α antibody (Cat # 558106) was from BD Bioscience. For intracellular cytokine staining, cells were stimulated with Cell Stimulation Cocktail (eBioscience, Cat # 00-4975-93) for 4 hours, followed with fixation and staining with Foxp3/transcription factor staining buffer set (eBioscience, Cat # 00-5523-00). Flow cytometry analyses were performed on a BD LSRII flow cytometer. Cell sorting was performed on a BD FACS ARIA II high-speed cell sorter. Data was analyzed with FlowJo software. Statistical significance between groups was determined by Student’s t-test for pairwise comparison (*p* < 0.05).

### 2.4 qRT-PCR analysis

Total RNA was extracted with a RNeasy Plus kit (Qiagen, Cat # 74134) and reverse transcribed using an iScript kit (Biorad, Cat # 1708891). qRT-PCR was performed using SsoAdvanced Universal SYBR Green Supermix (Biorad Cat # 1725275) on a Biorad CFX96 machine. Expression data were normalized to *Rpl32* mRNA levels. The data were calculated as 2^(Ct(RPL32-gene of interest))^ to compare experimental groups to controls, and are presented in arbitrary units. Students’ t test (*p* < 0.05) for pairwise comparison was used to determine significance. Primer sequences are listed in **Table 1**. Whenever possible, primers were intron-spanning, such that amplification is feasible on complementary DNA.

**Table 1:** Primer sequence for qPCR.

Primer	Reverse	Forward
*Rpl32*	TTGTGAGCAATCTCAGCACA	GGGAGCAACAAGAAAACCAA
*Il17re*	CTCTGGAAGGATGCTGGTGT	GCCTACCGTGTGGATAAACG
*Foxp3*	CGTGGGAAGGTGCAGAGTAG	ACTGGGGTCTTCTCCCTCAA
*Rorc*	TGCAGGAGTAGGCCACATTAC	CCGCTGAGAGGGCTTCAC

### 2.5 iTreg differentiation *in vitro*

Naïve T cells were purified from C57BL/6 mouse spleen using EasySep™ Mouse Naïve CD4+ T Cell Isolation Kit (Cat # 19765) from STEMCELL Technologies. 6 x 10^5^ Naïve T cells were cultured with anti-CD3 monoclonal antibodies (2 μg/mL, coated) and anti-CD28 monoclonal antibodies (2 μg/mL, soluble) in a 24-well plate. To induce differentiation of Treg cells, TGF-β (10 ng/mL, PeproTech) and IL-2 (20 ng/mL, PeproTech) were added to the mouse cell culture media with 10% FBS for 72 h, followed by the treatment of 5 ng/mL IL-2 with or without 50 ng/mL IL-6 for 24h. Cells were harvested for q-RT-PCR analysis.

### 2.6 Single-cell RNA-Seq data processing and analysis

Bone marrow cells from *Foxp3-YFP-Cre^+^/Il17ra* ^F/F^ (KO) and *Foxp3-YFP-Cre^+^/Il17ra* ^F/+^ (WT control) mice were adoptively transferred into 6-8-week-old *Cdx2-Cre^+^*/*Apc* ^F/+^ mice. Bone marrow recipient mice were then sacrificed at 5 months of age, and their mesenteric lymph nodes (MLN) and tumors were digested with 1 mg/ml collagenase IV for 20 minutes. Cells were filtered with a 70-μm cell sieve and stained with Live/Dead fixable exclusion dye, followed by fluorochrome-conjugated antibodies in PBS with 2% fetal bovine serum (FBS) and 1mM EDTA. Live/CD45^+^/CD3^+^/CD4^+^/YFP^+^ cells were sorted and loaded for capture using the Chromium System with single-cell 3’ reagent kit v2 from 10X Genomics. After capture and lysis, cDNA synthesis and amplification were performed according to manufacturer protocol. Amplified cDNA was used to construct the Illumina sequencing library and sequenced on the NovaSeq 6000. Illumina basecall (.bcl) files were converted to FASTQ using bcl2fastq from the CellRanger pipeline. Fastq files were aligned to the mm39 mouse reference genome and transcriptome and cell-counts matrices were generated using 10x Genomics CellRanger v7.0 with default parameters. Single-cell RNA sequencing cell-counts matrices were analyzed using Seurat v4.3 (27) with RStudio v4.2. A merged Seurat object was generated, and QC was performed; retaining cells with >= 500 genes and <= 30% reads mapping to mitochondrial genes. Gene expression was normalized, scaled and dimensionality reduction was performed. UMAPs of clusters, experimental conditions and IL-17 receptor gene expression were generated using Seurat’s FeaturePlot function and the R package ggplot2. Dotplots, barplots and heatmaps were generated using the R package ggplot2. Differential gene expression was determined using Seurat’s FindMarkers function (adjusted *p*-value < 0.05, log2 Fold Change >= 0.25, min.pct >= 0.1).

### 2.7 Analysis of RNA velocity in scRNAseq data

RNA velocity in CRC Tregs from the MLN and tumor were analyzed using Velocyto (28). The output from CellRanger was analyzed using the run10x function from Velocyto with the GenCode mm39 primary assembly transcriptome and mm39 repeat masker file from the UCSC genome browser with all parameters as default. Using the loom file output from Velocyto, the trajectory UMAP of merged data was generated using the scVelo stochastic model (29) with the python modules scanpy v1.9.6 and scvelo v0.2.5.

### 2.8 Pathway enrichment analysis

Genes which were significantly differentially expressed (log2 Fold Change >= 0.25, min.pct >= 0.1, adjusted *p*-value < 0.05) were subject to pathway enrichment analysis using the R packages gprofiler2 (30), clusterProfiler (31), and enrichplot. Enrichment was conducted using the Gene Ontology database as the pathway source. Pathways with a fold enrichment >= 2 were rank sorted based on their significance (adjusted *p*-value < 0.05) and the top 20 enriched pathways were plotted as a barplot using ggplot2. The gene network of a subset of pathways was generated using the cnetplot function from enrichplot along with ggplot2.

### 2.9 Analysis of human scRNAseq datasets

All human scRNAseq datasets were obtained from the Gene Expression Omnibus (GEO). Pancreatic cancer data (32) was obtained from the GEO accession number GSE155698. Melanoma data (33) was obtained from GSE239750. Triple-negative breast cancer data (34) was obtained from GSE169246. Liver cancer data (35) was obtained from GSE98638. Non-small cell lung cancer data (36) was obtained from GSE99254. Colorectal cancer data (37) was obtained from GSE178341. Counts-matrices were merged, gene expression was normalized and scaled, and Tregs were subset according to the original author specification, where available, with confirmation by expression of *Foxp3* in all cells. Conditions for each dataset (i.e. tumor, adjacent normal tissue, peripheral blood, etc.) were determined based on the original manuscripts’ specifications. The tumor Treg signature was generated by calculating the mean and standard error of the mean for the sum value of genes *GADD45B, RGS2, NR4A1, TNFRSF9, CCL5, CXCR6, CREM, TNFRSF18*, and *TNFRSF4*. The heatmap of the individual genes comprising the Treg signature was generated using ggplot2. Differential gene expression was determined using Seurat’s FindMarkers function (adjusted *p*-value < 0.05, log2 Fold Change >= 0.25, min.pct >= 0.1).

### 2.10 Bulk RNA sequencing and analysis

Similar to single-cell sequencing described above, MLN and tumor-infiltrating Treg cells were sorted, and their RNA was isolated using Qiagen RNeasy Plus Mini Kit. cDNA libraries were generated for poly-A selected RNA using the Illumina TruSeq mRNA Library Kit. 100 bp paired end reads were sequenced on the Illumina NextSeq 550 v2.5. Paired end reads in fastq format were aligned to the mm39 mouse genome and transcriptome using Hisat2 (38). Transcripts were assembled, gtf files for all conditions were merged, and read coverage tables were generated using StringTie (39) v2.2.1. Read counts were extracted using the prepDE python script from Stringtie, and normalization, calculation of FPKM values, and differential expression analysis were performed using the R package DESeq2 (40) (adjusted *p*-value < 0.05). Bar plots, heat scatter plots and heatmaps were generated using ggplot2 and coefficient of determination was calculated using the stats R package. Differential isoform usage, and splicing event enrichment were analyzed using the R packages DexSeq and IsoformSwitchAnalyzeR (41) Differential isoform usages were calculated with a dIF absolute value >= 0.1 and an adjusted *p*-value < 0.05. Splicing event enrichment was calculated using the extractSplicingEnrichmentComparison function from the R package isoformswitchanalyzeR with default parameters (alpha=0.05, dIFcutoff=0.1).

### 2.11 Statistical analysis

Statistical methods for RNA sequencing data analyses are described in previous sections. Otherwise, data were analyzed by the Student’s t-test for pair-wise comparisons. p values less than 0.05 were considered significant.

## 3 Results

### 3.1 Tumor-infiltrating Tregs form unique clusters and are activated and immunosuppressive in colorectal tumors

**Figure 1:**
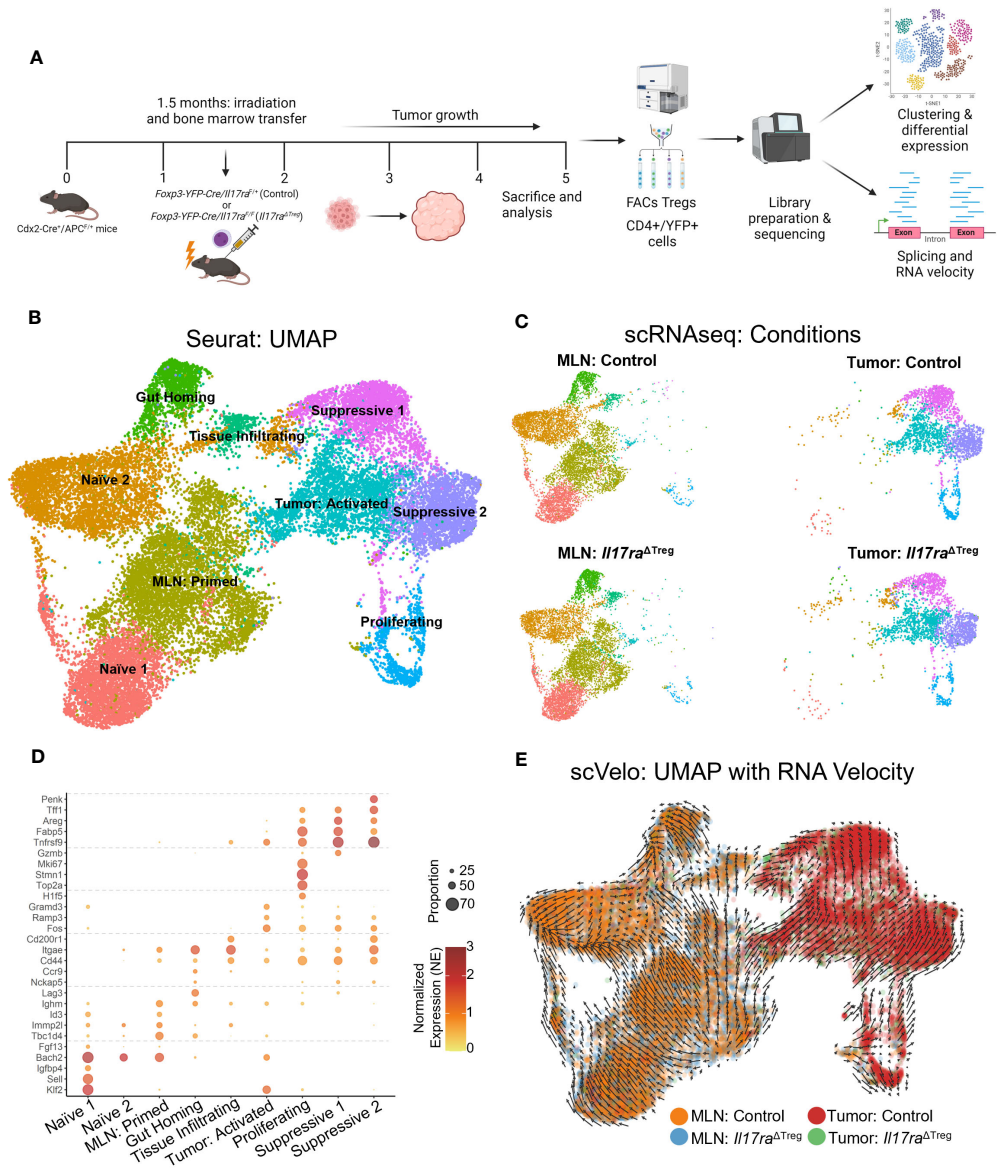
Tumor-infiltrating Tregs form distinct clusters compared to MLN Tregs. **(A)** Bone marrow cells from *Il17ra* ^ΔTreg^ and control mice were adoptively transferred into 6-8-week-old *Cdx2-Cre^+^*/*Apc* ^F/+^ mice. Bone marrow recipient mice were then sacrificed at 5 months of age, and their mesenteric lymph nodes (MLN) and tumors were dissociated and FACS purified for live/CD45^+^/CD3^+^/CD4^+^/YFP^+^ cells. These cells were then subjected to single-cell RNA sequencing analysis. **(B)** Uniform manifold approximation and projection (UMAP) with clustering analysis for Treg scRNAseq data. Tregs isolated from the four conditions were merged and analysis revealed a total of 9 clusters across the datasets. **(C)** UMAPs of Treg scRNAseq data split by conditions. **(D)** Gene markers for each of the 9 scRNAseq clusters. Two naïve Treg subsets, primed, gut homing and tumor-infiltrating subsets were observed in the MLN, as well as tumor activated, proliferating and tissue-resident suppressive Tregs were observed within the tumor. **(E)** Analysis of RNA velocity from scRNAseq datasets using Velocyto. Trajectory UMAP of merged data was generated using the stochastic model from scvelo.

IL-17 family cytokines signal to diverse cellular targets, including epithelial cells, fibroblasts, and myeloid cells (15, 18). A direct signaling of IL-17 on Tregs is unknown, but we contend that it may be important for the function of Tregs and development of tumors. To this end, *Foxp3-YFP-Cre* mice were crossed to *Il17ra* ^F/F^ mice to specifically ablate IL-17RA in Tregs (*Foxp3-YFP-Cre* ^+^/*Il17ra^F/F^* mice, hereby referred to as *Il17ra* ^ΔTreg^ mice). YFP transgene under the control of the *Foxp3* promoter also serves as a marker of Tregs *in vivo* and was used to gate these cells for flow cytometry and cell sorting. To investigate how *Il17ra* KO impacts Tregs in CRC, we adoptively transferred bone marrow cells from *Foxp3-YFP-Cre* ^+^/*Il17ra^F/+^* (control) and *Il17ra* ^ΔTreg^ mice into recipient mice that develop sporadic CRC (*Cdx2-Cre^+^*/*Apc* ^F/+^). These bone marrow recipient mice are herein referred to as “control CRC” and “*Il17ra* ^ΔTreg^ CRC” mice, respectively. 3.5 months after bone marrow transfer, control and *Il17ra* ^ΔTreg^ CRC mice were sacrificed, and Tregs were purified by cell sorting from the mesenteric lymph node (MLN) and colorectal tumors and subjected to single-cell RNA sequencing (scRNAseq) (**Figure 1A**). Unsupervised clustering of MLN and tumor Tregs revealed a total of 9 clusters within the merged dataset (**Figure 1B**). MLN Tregs were predominantly comprised of naïve, primed, and gut homing/tissue-infiltrating Tregs, while colorectal tumor contains tumor activated Tregs, proliferating Tregs and two tissue-resident immunosuppressive Treg clusters (**Figure 1C**). Treg clusters were named based on analysis of differentially expressed genes (**Figure 1D**): Naïve and primed Tregs in the MLN were marked by the expression of the transcription factor *Bach2*, which represses effector T cell programs and is required for maintaining naïve T cell state (**Figure 1D**) (42, 43). Following priming, Bach2 was downregulated, along with upregulation of activation markers *Lag3* (44), *CD44* (45), as well as gut homing signatures *Ccr9* (46) and *Itgae* (**Figure 1D**) (47, 48). In the tumor, Treg clusters displayed a gradual increase in the expression of secondary costimulatory receptor OX40 (*Tnfrsf9*) (49) and tissue resident Treg markers *Areg*, *Tff1* and *Penk* (**Figure 1D**) (50–52). The proliferating cluster, comprised of Tregs primed in the MLN and Tregs activated in the TME, was marked by expression of *Mki67*, *Stmn1* and *Top2a* (**Figure 1D**) (53–55). These data indicate distinct clustering of tumor infiltrating Tregs compared to Tregs in peripheral lymphoid tissues. To validate the progressive transcriptomic shift in Treg identity between clusters, we analyzed RNA velocity in the Treg subpopulations. RNA velocity infers the temporal dynamics of gene expression within a population based on the proportion of spliced versus unspliced transcripts, under the assumption that steady state cell identity will result in a higher proportion of spliced transcripts compared to cells undergoing dynamic shifts in cell identity. For example, differentiation or activation, will display an increased proportion of unspliced transcripts, as transcription precedes mRNA processing (29). RNA velocity revealed a global transcriptomic shift beginning with the naïve and primed Treg clusters in the MLN towards the proliferating and activated Treg subpopulations in the tumor, and finally towards the immunosuppressive tissue resident Treg clusters in the tumor (**Figure 1E**). These data demonstrate that naïve Tregs in the MLN undergo priming and transcriptomic changes allowing for their gut homing and infiltration of colon tumors. Once in the tumor, they are activated, proliferate, and undergo further maturation, allowing for their immunosuppressive function within the tumor microenvironment (TME).

### 3.2 IL-17 co-receptors are restricted to tumor-infiltrating Tregs

**Figure 2:**
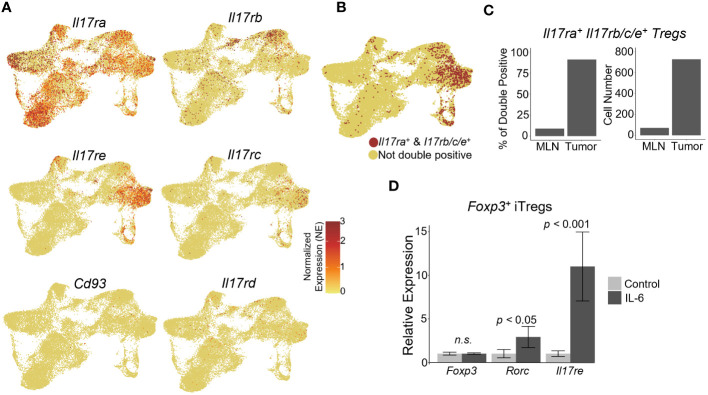
Tumor-infiltrating Tregs express IL-17 co-receptors necessary for sensing IL-17. **(A)** IL-17 receptor subunit gene expression analysis within scRNAseq data. *Il17ra* was expressed in both the MLN and Tumor subsets. *Il17rb, Il17rc*, and *Il17re* were enriched in the tumor. *Il17re* and *Il17rc* were expressed predominantly in suppressive cluster 1 and the proliferating cluster within the tumor, while *Il17rb* was expressed predominately in the suppressive cluster 2 and the tumor-infiltrating cluster. No robust expression of *Il17rd* or *Cd93* were observed. **(B)** UMAP of cells expressing functional Il17 receptor heterodimers with *Il17ra*: *Il17ra/rb, Il17ra/rc, Il17ra/re*. **(C)** Proportion and normalized cell number of double positive cells in the MLN and tumor. **(D)** Naïve CD4^+^ T cells were isolated from mouse spleens with a naïve T cell isolation kit from STEMCELL, and differentiated into iTreg cells for 3 days. Differentiated iTregs were subsequently stimulated with 50 ng/ml IL-6 for 24 hours, and their RNA were subjected to qRT-PCR analysis (*p*-value < 0.05, n= 3, student’s t test).

IL-17 mediated tumor-associated inflammation has been shown to promote tumor development in the gut (20, 22, 23). The IL-17 receptor is comprised of 5 currently identified members (14). IL-17RA forms heterodimers with the other IL-17 co-receptors to bind IL-17 ligands. For IL-17 to be able to directly signal to Tregs, these cells should express both IL-17RA and its co-receptors. Thus, we analyzed the expression of IL-17 receptor subunits within the Treg subpopulations. *Il17ra* was ubiquitously expressed throughout the Treg clusters in both the MLN and tumor (**Figure 2A**). IL-17RA interaction with IL-17RC allows binding of IL-17A, IL-17F, or the IL-17A/F heterodimer (56, 57). IL-17RA/IL-17RB heterodimer binds IL-17B and IL-17E, and IL-17RE interacts with IL-17RA to bind IL-17C (58, 59). Both *Il17re* and *Il17rc* were expressed within the proliferating and suppressive 1 clusters of tumor Tregs, whereas *Il17rb* was expressed within suppressive cluster 2 of the tumor Tregs (**Figure 2A**). IL-17RD, which can heterodimerize with IL-17RA to bind IL-17A, was not reasonably expressed in any clusters of the Treg scRNAseq data (**Figure 2A**) (60, 61). Additionally, the noncanonical IL-17 receptor gene *Cd93*, whose protein acts as a receptor for IL-17D, was not reasonably expressed in the Treg data (**Figure 2A**) (16). Examining the cells which could form functional IL-17 receptor heterodimers, based on co-expression of *Il17ra* with either *Il17rb/c/e*, suggested that while *Il17ra* was expressed throughout the Treg clusters, the tumor Treg clusters, specifically, suppressive 1, 2, and proliferating clusters, predominantly represented the cells capable of expressing the necessary co-receptors for IL-17 signaling (**Figures 2B, C**). We hypothesized that IL-17 receptors may be upregulated specifically in tumor infiltrating Tregs due to inflammatory signals in the TME. Amongst inflammatory cytokines within the TME, IL-6 is highly expressed in colorectal tumors and has been shown to promote Th17 differentiation and IL-17RE expression (24). To investigate whether IL-6 could contribute to the expression of *Il17re* on Tregs, we stimulated *in vitro* differentiated iTregs with IL-6. While *Foxp3* mRNA levels were unchanged following stimulation, we observed a significant *Il17re* upregulation (*p* < 0.001) as well as a modest, but statistically significant increase in the expression of *Rorc* (encoding RORγt) (*p* < 0.05) (**Figure 2D**). Thus, IL-6 secretion by inflammatory cells within the TME, may lead to the upregulation of co-receptors necessary for IL-17 signaling on Tregs.

### 3.3 IL-17 enhances Treg maturation and suppression gene signatures

**Figure 3:**
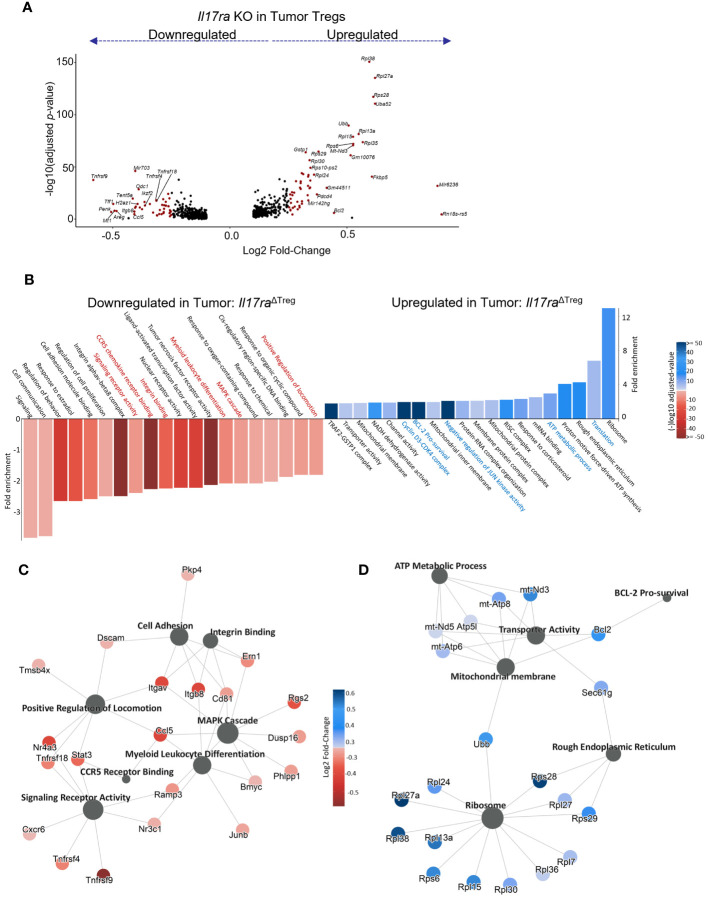
IL-17 promotes suppression gene signatures in tumor-infiltrating Tregs. **(A)** Volcano plot of statistically significant (log2FC >= 0.25, adjusted *p*-value < 0.05, min.pct >= 0.1) differentially expressed genes in scRNAseq data between *Il17ra^ΔTreg^* and control tumor-infiltrating Tregs. **(B)** Gene Ontology (GO) pathway enrichment analysis of genes significantly up or downregulated by *Il17ra* KO in tumor Tregs. **(C, D)** Gene network plots of a subset of pathways significantly enriched amongst the down **(C)** and upregulated **(D)** DEGs by IL-17 signaling.

As the tumor-infiltrating Tregs represented the population capable of responding to IL-17 signaling, we next analyzed the genes differentially expressed between WT vs *Il17ra* KO Tregs from tumors of control and *Il17ra* ^ΔTreg^ CRC mice (**Figure 3A**). Pathway enrichment analysis of genes significantly differentially expressed (log2 fold change >= 0.25, adjusted *p*-value < 0.05, min.pct >= 0.1) revealed that *Il17ra* KO tumor Tregs exhibited an increase in pathways related to translation, metabolism, and survival, with a decrease in pathways related to T cell function, namely leukocyte differentiation, integrin binding, and chemokine signaling (**Figure 3B**). Several highlighted pathways in *Il17ra* KO tumor Tregs (**Figures 3C, D**) show the upregulation of multiple ribosomal subunits, mitochondrial genes related to oxidative phosphorylation, and pro-survival signaling (**Figure 3D**). Oxidative phosphorylation has been shown to be crucial for Treg survival, especially in low glucose, high lactate environments, such as the intestinal tract (62, 63). *Il17ra* KO tumor Tregs also displayed a downregulation in established IL-17 signaling-related genes which are important for Treg function, including *Junb* (64, 65)*, Stat3* (66, 67) and genes related to MAPK cascade (68, 69) (**Figure 3C**). Secondary costimulatory molecule genes upregulated following T cell activation, *Tnfrsf9* (4-1BB) (70, 71), *Tnfrsf4* (OX40) (49, 72, 73), and *Tnfrsf18* (GITR) (74) were also downregulated in *Il17ra* KO Tregs in the tumor (**Figure 3C**). Also downregulated were genes essential to Treg tissue homing and suppressive function including the αVβ8 integrin receptor genes *Itgav* and *Itgab* (75, 76), as well as CD81 (77) and the transcription factor *Nr4a3* (78) (**Figure 3C**). A full list of DEGs can be found in **Supplementary Table 1**. We also analyzed the subset of Tregs which express IL-17RA co-receptor genes *Il17rb/rc/re* (**Supplementary Figure 1A**) and found a similar pattern of upregulated and downregulated genes (**Supplementary Figure 1B**). Also included amongst these DEGs were suppression-associated genes *Ctla4* (79), *Gzmb* (80), *Ikzf2* (encoding the transcription factor Helios) (81) and *Tnfrsf18* (GITR) (82), tissue-resident Treg markers *Areg, Penk, Tff1, and Odc1*, and activation/maturation-associated genes *Tnfrsf4, Tnrfsf9, Mt1*, and *Got1* (**Supplementary Figures 1B, C**). These data suggest that while *Il17ra* KO Tregs display an increased capacity for survival and mitochondrial function/metabolism, their function as suppressors of inflammation is reduced.

### 3.4 IL-17 signals to Tregs to control CRC development

**Figure 4:**
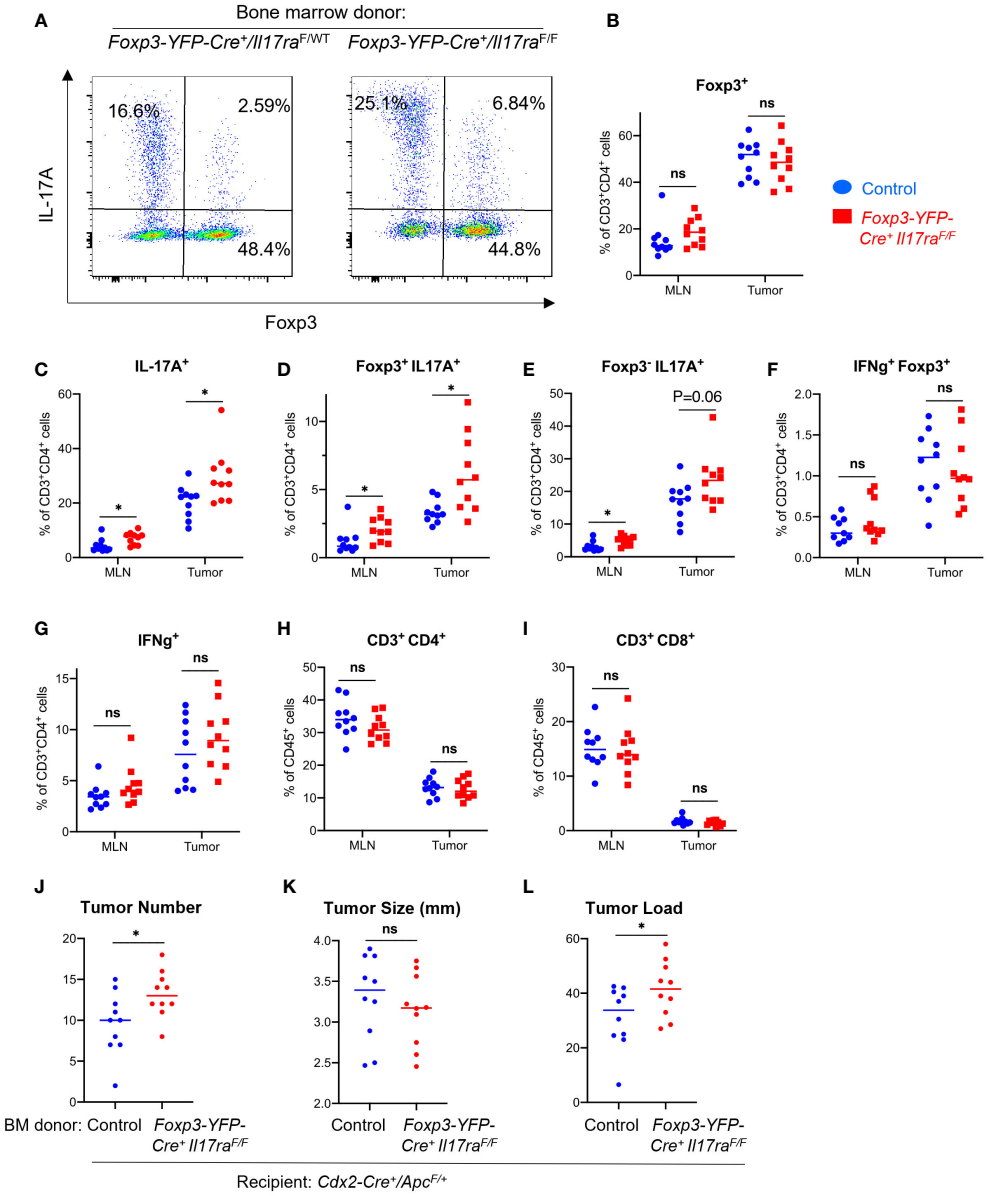
IL-17 signaling to Tregs controls Th17 population and inhibits CRC development. *Cdx2-Cre^+^*/*Apc* ^F/+^ mice received adoptive transfer of bone marrow cells from *Foxp3-YFP-Cre^+^/Il17ra* ^F/F^ (*Il17ra* ^ΔTreg^) and *Foxp3-YFP-Cre^+^/Il17ra* ^F/+^ (control) mice, and were sacrificed at 5 months of age. MLN and tumor tissues were dissociated and subjected to flow cytometry analysis. **(A–I)** MLN and tumor cells from indicated mouse populations were stimulated with PMA/ionomycin in the presence of brefeldin A and monensin, and stained for intracellular cytokines and Foxp3. Stained cells were gated on the live/CD45^+^ population, n=10. **(J–L)** Colorectal tumors were measured with a caliper, and their numbers and average tumor diameters are shown, n=10. Tumor load was calculated as the sum of all tumors’ diameters of each animal. *p<0.05, Students’ *t* test. ns, not significant.

A small fraction of Tregs in the tumor also upregulated IL-17, indicating plastic functions of these cells (**Figures 4A, D**), although the total number of FOXP3^+^ Tregs did not significantly change upon ablation of IL-17RA (**Figure 4B**). Consistent with our observation that IL-17 promotes Treg suppressive function, ablation of IL-17RA in Tregs leads to an increased proportion of CD4^+^ T cells that express IL-17 (**Figure 4C**). These IL-17 producing cells are either Foxp3^+^ or Foxp3^-^, suggesting both enhancement in Th17 population and increased Treg plasticity (**Figures 4D, E**). We did not observe a noticeable change in the proportions of CD4^+^ T cells that produce IFN-γ (**Figures 4F, G**), or changes in the proportion of CD4 or CD8 cells among all CD45^+^ cells in the tumor (**Figures 4H, I**). Importantly, ablation of IL-17 signaling in Tregs resulted in increased tumor number and tumor load in mice, demonstrating a previously unknown tumor-inhibitory role of IL-17R through its function in Tregs (**Figures 4J–L**).

### 3.5 IL-17 promotes a unique “tumor signature” in Tregs

**Figure 5:**
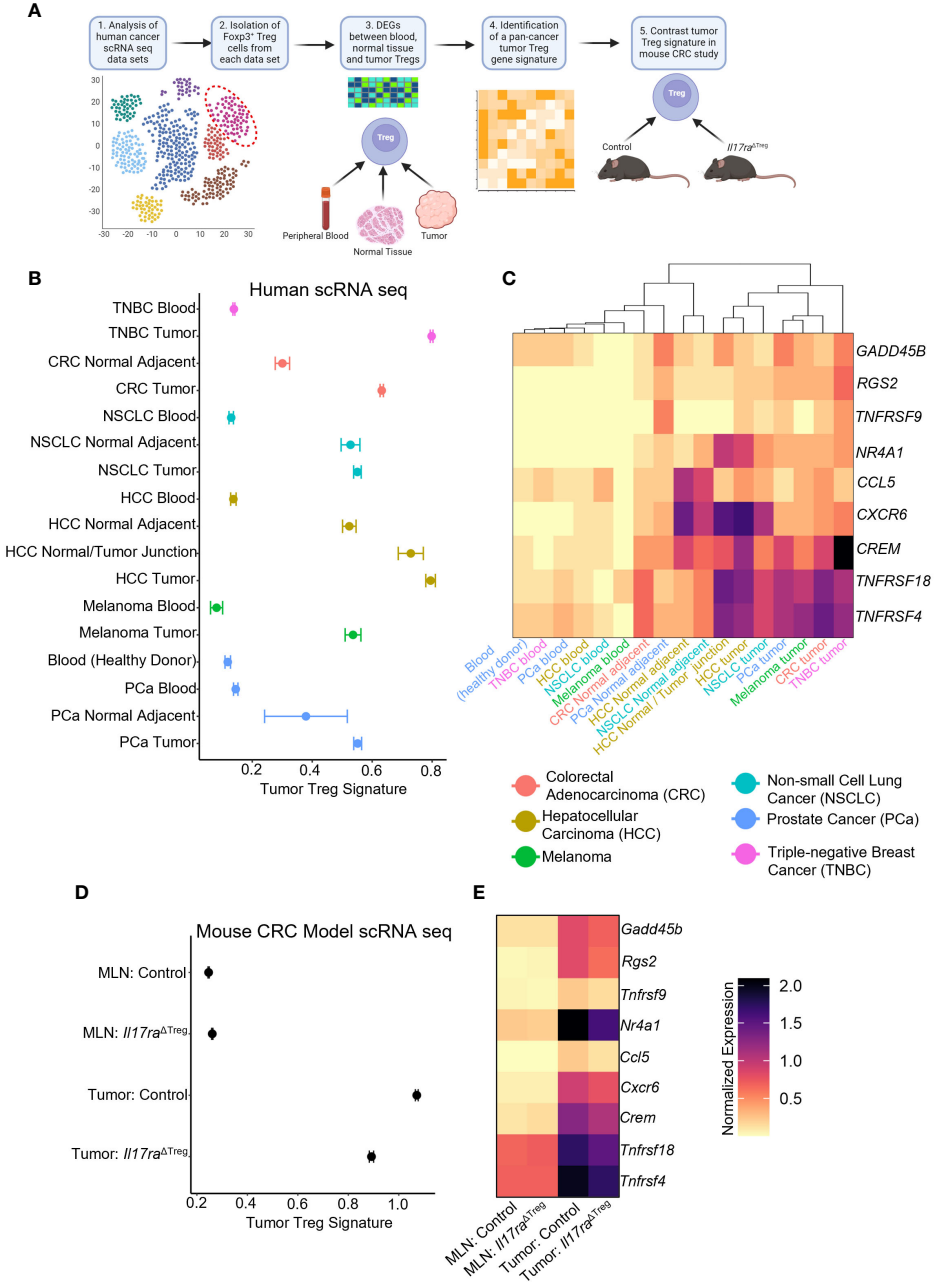
IL-17 promotes a “tumor Treg signature” in cancers. **(A)** Scheme of tumor Treg signature development. Human cancer scRNAseq datasets were analyzed for populations of Tregs. Treg cells were subset from each human cancer dataset based on the original authors’ cell-type assignments, where available, and confirmation of expression of *FOXP3* in all immune cells. Differential expression analysis was performed between conditions (peripheral blood, normal tissue, tumor samples). Genes were filtered to retain those which were enriched in the tumor conditions (log2 fold-change >= 0.25, adjusted *p*-value < 0.05, min.pct >= 0.1) relative to their associated control conditions in all data sets to obtain a pan-cancer/tissue gene signature specific to tumor infiltrating Tregs. This signature was used for comparative study in the mouse CRC datasets. **(B)** Dotplot of Tumor Treg signature score for Tregs isolated from human cancer patient scRNAseq datasets. **(C)** Heatmap of the individual genes comprising the composite tumor Treg signature, for each of the human scRNAseq datasets with hierarchical clustering of datasets by heatmap dendrogram. **(D)** Tumor Treg signature in each of the four scRNAseq conditions from control and *Il17ra* ^ΔTreg^ Tregs isolated from the MLN and tumor. **(E)** Heatmap of individual genes comprising the tumor Treg score. All genes between tumor control and tumor *Il17ra* ^ΔTreg^ conditions were significant (log2 fold-change >= 0.25, adjusted *p*-value < 0.05, min.pct >= 0.1).

To further investigate how IL-17 signaling impacts Treg function, we analyzed human cancer patient scRNAseq datasets containing FOXP3^+^ Treg populations (32–37) (**Figure 5A**). Analysis of Treg populations within human tumors, normal adjacent tissues, and peripheral blood samples revealed genes associated with activation, differentiation, and suppressive function in the tumor. From these genes we created a tumor Treg signature which identified Tregs from the blood, adjacent tissue, and tumor that can be distinguished based on our gene signature (**Figure 5B**). The gene signature was comprised of genes associated with T cell activation, including *RGS2* (83), *Gadd45b* (84), *TNFRSF9* (4-1BB) (70, 71)*, TNFRSF4* (OX40) (49, 72, 73), and *TNFRSF18* (GITR) (74). Also included were the Treg differentiation transcription factor *NR4A1* (85), tumor Treg associated gene *CCL5* (86), as well as immunosuppression-related genes *CREM* (87) and *CXCR6* (88). A heatmap of the individual genes that comprised the Treg signature, along with unbiased hierarchical clustering of the tissue samples, grouped all tumor, adjacent normal tissue, and peripheral blood samples, irrespective of the cancer/tissue type of origin (**Figure 5C**). An examination of the mouse homolog genes in the scRNAseq datasets of MLN and tumor Tregs from control and *Il17ra* ^ΔTreg^ CRC mice revealed a similar pattern, with low expression in both the control and *Il17ra* ^ΔTreg^ CRC MLN Tregs, and high expression in the control tumor Tregs. Importantly, the *Il17ra* KO tumor Tregs had a reduced tumor Treg signature and were shifted towards the lower levels found in the MLN conditions (**Figure 5D**). A heatmap of the individual genes comprising the tumor Treg signature shows each gene is downregulated by *Il17ra* KO in the tumor Tregs (log2 fold-change >= 0.25, adjusted *p*-value < 0.05), but were not significantly altered by KO in Tregs in the MLN (**Figure 5E**). These data suggest a Treg gene signature which can unbiasedly distinguish blood, normal tissue, and tumor Tregs, regardless of cancer/tissue type. This gene signature associated with tumor Tregs is conserved between mouse and human and is significantly reduced by ablation of IL-17RA in these cells.

### 3.6 IL-17 promotes RNA splicing that is important for Treg function

**Figure 6:**
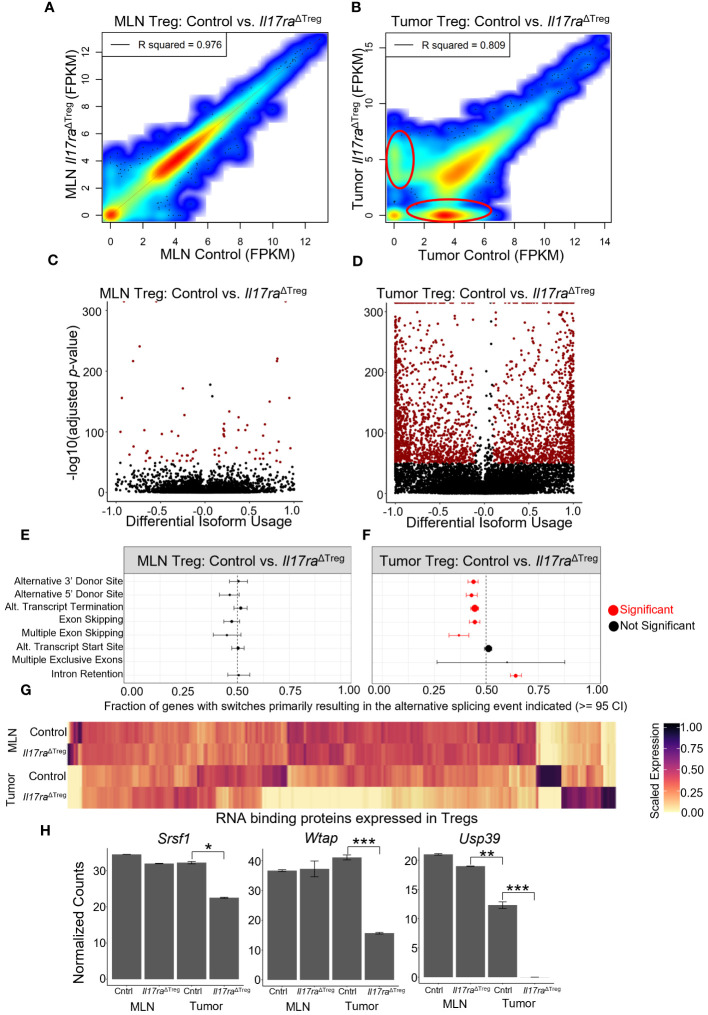
Treg-specific IL-17RA signaling modulates RNA splicing. **(A, B)** Scatter heat plot of FPKM values from bulk RNA sequencing (bulkRNAseq) data between control and *Il17ra* ^ΔTreg^ in the MLN **(A)** and tumor **(B)** analyzed using DESeq2. **(C)** Volcano plot of significant transcript isoform switches occurring between MLN WT and KO compared to **(D)** significant transcript isoform switches occurring between tumor WT and KO (highlighted in red: differential isoform usage >= 0.1, -log10(adjusted *p*-value) >= 50). **(E, F)** Analysis of splicing events occurring in transcripts between WT and KO conditions in the MLN **(E)** and tumor **(F)**. Significant events highlighted in red (>= 95 confidence interval). **(G)** Heatmap of RNA binding protein genes (annotated as ‘RNA splicing’ by gene ontology) expressed in MLN or tumor Tregs. **(H)** Bar plots of significantly differentially expressed RNA splicing regulators *Srsf1, Wtap*, and *Usp39* in bulk RNA sequencing of WT and KO conditions in the MLN and tumor. Adjusted p-values were determined using DESeq2 (*p<0.05, **p<0.001, ***p<0.0001).

Analysis of RNA velocity in mouse Treg scRNAseq data revealed splicing changes resulting from Treg activation and tumor infiltration (**Figure 1E**). However, conventional techniques employed for scRNAseq limits the capacity for full or deep analysis of splicing due to the high rate of dropout, low sequencing depth and low coverage due to 3’ end sequencing of RNA (89, 90). Therefore, to investigate if *Il17ra* KO influences alternative splicing in Tregs, we isolated CD4^+^ FOXP3^+^ cells from the MLN and colon tumors and performed bulk RNA sequencing. An analysis of gene expression levels (FPKM) between control and *Il17ra* KO Tregs in the MLN showed a strong correlation between conditions (R^2^ = 0.976) (**Figure 6A**). However, in the tumor, the correlation between control and *Il17ra* KO was reduced (R^2^ = 0.809), with visible populations of differentially expressed genes between the two conditions (circled in red) (**Figure 6B**). These findings agree with our observation, through single-cell sequencing, that response to IL-17 signaling is limited to Tregs infiltrating the tumor environment, and that changes in gene expression profiles and Treg functions are tumor-specific. An analysis of differential isoform usage demonstrated that while few significant changes occurred in the MLN (-log10 adjusted *p*-value) there were robust isoform switches occurring between control and *Il17ra* KO Tregs in the tumor (**Figures 6C, D**). Consistent with our previous findings from scRNAseq, these data demonstrate that compared to Tregs in the MLN, where Tregs lack the necessary co-receptors of IL-17RA, tumor-infiltrating Tregs exhibit significant changes in isoform usage when Treg-specific *Il17ra is* knocked out. We next analyzed enrichment of splicing events between *Il17ra* WT and KO Tregs in the MLN and tumor. We found that while no significant changes were observed between *Il17ra* WT and KO Tregs in the MLN (**Figure 6E**), in the tumor, *Il17ra* KO Tregs exhibited significant reductions in alternative 3’ and 5’ donor sites, alternative transcription termination sites, exon/multiple-exon skipping, and an increase in intron retention, relative to control Tregs in the tumor (**Figure 6F**). As RNA binding proteins play an essential role in mRNA processing and generation of alternative isoforms (91), we analyzed the gene expression of known RNA binding proteins (RBPs) which were expressed in the Treg bulk RNAseq data. While in the MLN, RBP expression appears similar, in the tumor, *Il17ra* KO Tregs showed a substantial number of RBPs which were downregulated, and a smaller proportion which were upregulated, relative to control tumor Tregs (**Figure 6G**). Recently, several studies have identified RBPs which are key regulators of splicing in Tregs and essential for their function and immunosuppressive effects. In particular, the RBPs *Srsf1* (92), *Wtap* (93), and *Usp39* (94) were all significantly downregulated in *Il17ra* KO tumor Tregs (**Figure 6H**). Together, these data suggest that the downregulation of RBPs, in the absence of IL-17 signaling in tumor Tregs, leads to a reduction in alternative splicing that is important for Treg activation and function in the tumor.

## 4 Discussion

**Figure 7:**
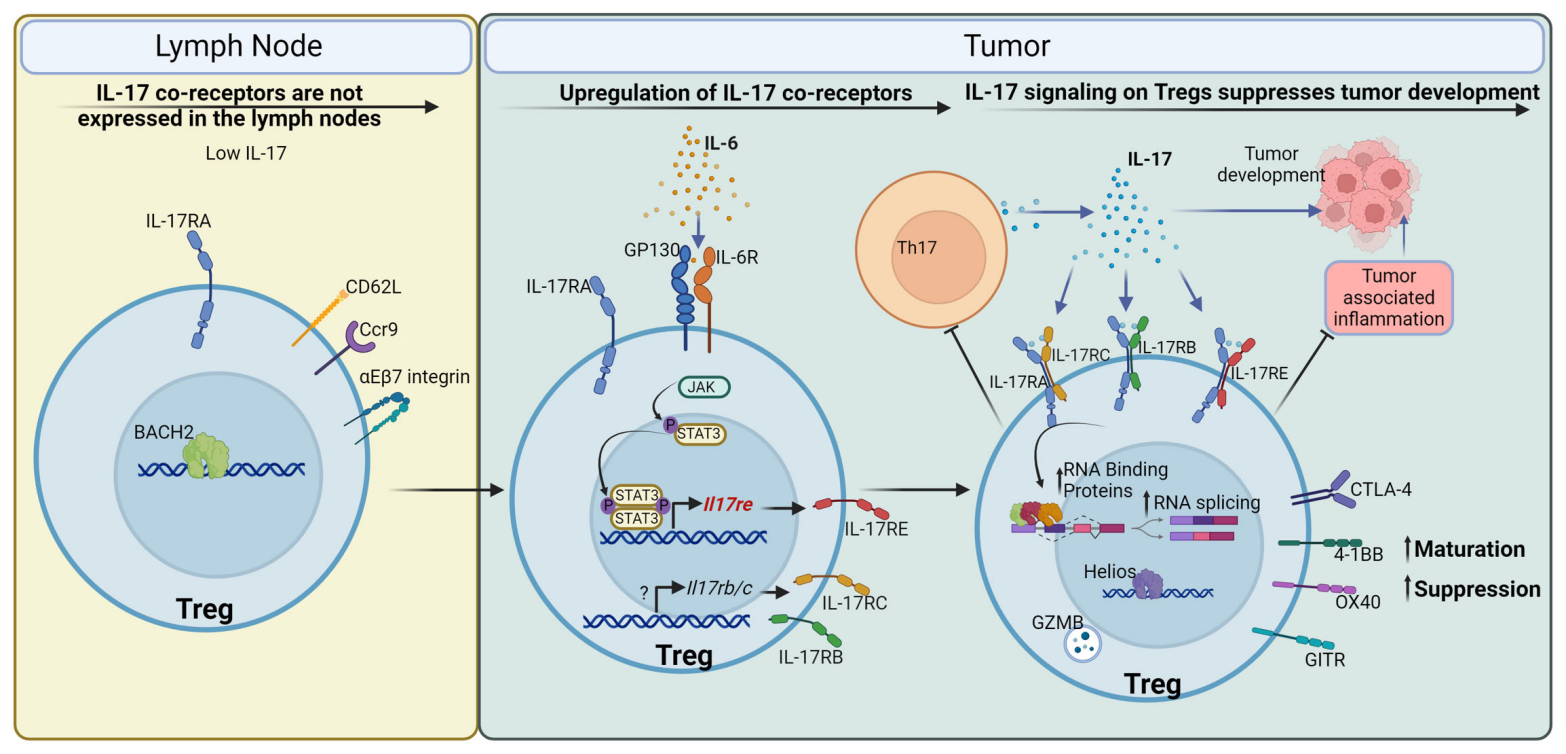
IL-17 promotes maturation and function of Tregs to control CRC development. Tregs in the mesenteric lymph node, where IL-17 is low, express *Il17ra* but do not express co-receptors required for IL-17 signaling. Upon entry to the tumor environment, Tregs are stimulated by IL-6 and other inflammatory cytokines to upregulate co-receptors for IL-17, such as IL-17RB, C, and E. IL-17 in turn signals to these Tregs and promotes their maturation and suppressive function. This is mediated by the upregulation of Treg surface receptors such as CTLA-4, 4-1BB (CD137), OX40 (CD134), and GITR, cytotoxicity effector Granzyme B, and transcription factor Helios. IL-17 also upregulates the expression of RNA binding proteins and facilitates RNA splicing in Tregs, which are important for Treg function. In turn, Tregs with enhanced activation and maturation markers suppress Th17 cells, leading to a reduction in IL-17 production, thus forming a negative regulatory loop to control IL-17-mediated inflammation. In the case of early-stage colorectal cancer, IL-17 mediated Treg maturation inhibits tumor-associated inflammation, and reduces tumor development in the gut.

In this study, we report a direct role of IL-17 in promoting Treg function in CRC (**Figure 7**). Contrary to what is known for the signaling of IL-17 to transformed epithelial cells (20, 22, 23), the IL-17/Treg pathway serves as a tumor suppressor, and its ablation resulted in increased tumor numbers and tumor load (**Figures 4J, L**). This observation is consistent with the known role of Tregs in downregulating tumor-promoting inflammation in early-stage colon cancer (8, 95–98). However, in late-stage cancers, a high intratumoral Treg : CD8 ratio typically signifies suppressed anti-tumor immunity and favors immune evasion of cancer (99). Treg number has been shown to correlate with poorer prognosis in multiple cancers (100–104). In addition to solid tumors, Tregs are also suggested to be a negative factor in the treatment of leukemias (105–107). It will be intriguing to also interrogate the interaction between IL-17 and Tregs in the setting of hematopoietic malignancies. Indeed, many therapies to prevent Treg suppressive function in the tumor environment are in development and in clinical trials (108). Our analysis on human tumor-infiltrating Tregs showed a unique tumor Treg signature that coincides with the mouse model of CRC, and downregulation of this signature by IL-17 in mice implies that direct IL-17/Treg signaling is plausible in human cancers. It remains to be interrogated if IL-17/Treg direct communication exacerbates immune evasion and/or sabotages cancer immunotherapy, and demonstrating this link will support the use of IL-17 blocking agents as adjuvant therapies for cancers.

Tregs are highly plastic and can co-express markers and secrete cytokines of conventional CD4^+^ T cell subsets (109). Tissue-resident Tregs express the Th2 transcription factor GATA3 in addition to FOXP3 (110). Additionally, while most IL-17 producing T cells are Th17, a small subset of FOXP3^+^/RORγt^+^ Tregs can produce IL-17A in both humans and mice (111–113). Our observation that Tregs upregulate IL-17 co-receptors following inflammatory cytokine stimulation also represents a form of plasticity in these cells. It is therefore plausible that peripheral Tregs, while devoid of IL-17 co-receptor expression, are attracted to the TME by chemokine gradients and aim at suppressing tumor associated inflammation/immunity. Once in the tumor environment, Tregs stimulated by cytokines, and possibly other environmental factors, upregulate IL-17 receptors. IL-17 in turn promotes further maturation of Tregs and enhances their function. It remains to be determined what signals *in vivo* activate IL-17 co-receptor expression in Tregs. However, we demonstrate that *in vitro*, iTregs respond to IL-6 by upregulating IL-17RE. On top of that, it is important to investigate if the TME is the only setting whereby Treg function is controlled by IL-17. Other promising targets include autoimmunity and chronic infection, where both IL-17 and Tregs play prominent roles. Indeed, in a model of MOG-immunization, a subset of Tregs were found to express both RORγt and CCR6 that is promoted by IL-6/STAT3 signaling in these cells (114). These “Tr17” cells also express IL-17RE, and are highly suppressive to antigen-specific Th1 and Th17 cells *in vitro* (114). The function of IL-17RE on these Tr17 cells is unknown. Investigating the IL-17/Treg signaling axis in these scenarios will provide new insight on the function and dysfunction of Tregs in human health and disease.

## Data availability statement

Raw and processed data from bulk RNA sequencing and single-cell RNA sequencing of CD4+ FOXP3+ Tregs from the tumor and mesenteric lymph node were deposited at the Gene Expression Omnibus (GEO). Bulk RNA sequencing data is available with accession number GSE262402 and single cell data is available with accession number GSE262405.

## Ethics statement

Ethical approval was not required for the studies on humans in accordance with the local legislation and institutional requirements because only commercially available established cell lines were used. The animal study was approved by University of Connecticut Health Center IACUC. The study was conducted in accordance with the local legislation and institutional requirements.

## Author contributions

WCT: Conceptualization, Writing – original draft, Data curation, Formal analysis, Methodology, Software. JC: Conceptualization, Data curation, Formal analysis, Methodology, Writing – original draft, Investigation, Validation. EVT: Formal analysis, Investigation, Writing – review & editing. XY: Formal analysis, Investigation, Writing – review & editing, Data curation. AM: Data curation, Formal analysis, Investigation, Writing – review & editing, Methodology. ATV: Writing – review & editing, Conceptualization, Resources, Supervision, Visualization. KW: Conceptualization, Supervision, Writing – review & editing, Funding acquisition, Project administration, Writing – original draft.
