# Supplementary material for: Interleukin-17 directly stimulates tumor infiltrating Tregs to prevent cancer development
Source: Front Immunol. 2024 Jun 14;15:1408710. doi: 10.3389/fimmu.2024.1408710 (PMC11211274; doi:10.3389/fimmu.2024.1408710)
Supplement: Supplementary file 1 [file DataSheet_1.pdf]

Supplemental Figure 1

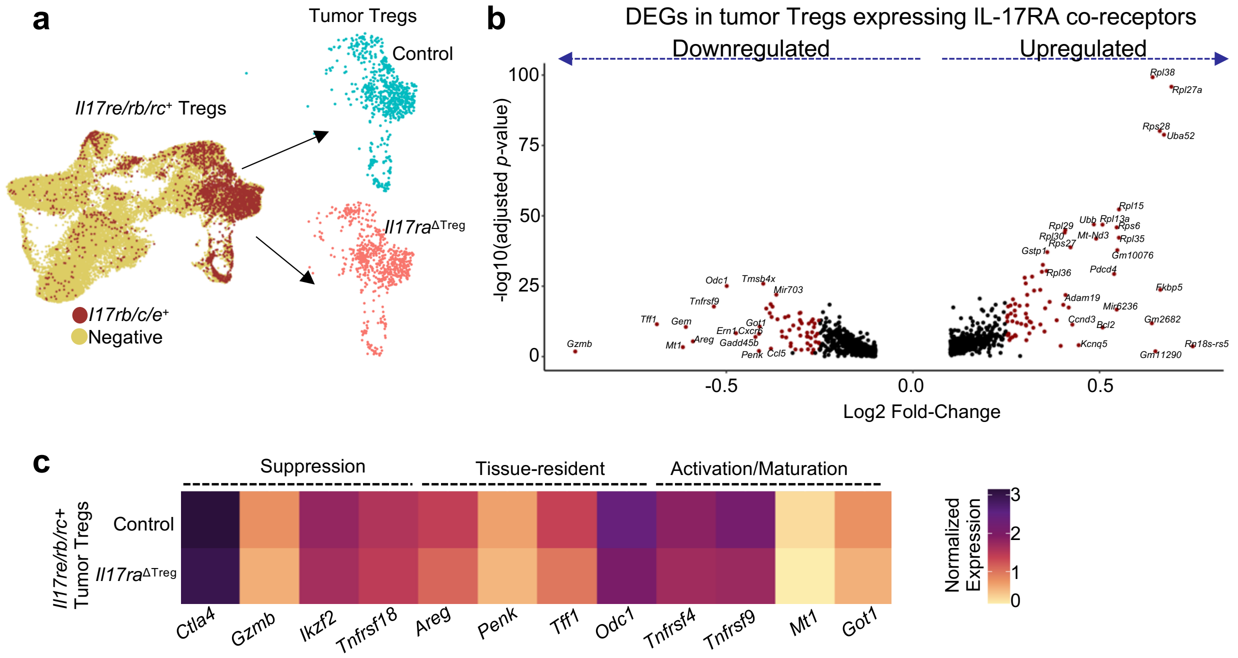


**Supplementary Figure 1: Loss of IL-17 signaling in IL-17 co-receptor positive Tregs reduced their expression of genes related to suppressive function, tissue infiltration and maturation.** (**a**) Schematic of selection criteria for IL-17 co-receptor positive cells. Tumor infiltrating Tregs which expressed *Il17re/rb/rc* in the tumor were selected for analysis of differentially expressed genes between *Il17ra*$^{\Delta Treg}$ and Control groups. (**b**) Volcano plot of genes significantly differentially expressed in *Il17re/rb/rc* positive Tregs (highlighted in red: log2 fold-change >= 0.25, adjusted *p*-value < 0.05). (**c**) Heatmap of genes related to suppressive function, tissue infiltration and Treg maturation between *Il17ra* KO and control tumor Tregs.
